# The timing of surgery after neoadjuvant radiotherapy influences tumor dissemination in a preclinical model

**DOI:** 10.18632/oncotarget.5931

**Published:** 2015-09-30

**Authors:** Natacha Leroi, Nor Eddine Sounni, Eva Van Overmeire, Silvia Blacher, Raphael Marée, Jo Van Ginderachter, François Lallemand, Eric Lenaerts, Philippe Coucke, Agnès Noel, Philippe Martinive

**Affiliations:** ^1^ Laboratory of Tumor and Development Biology, Groupe Interdisciplinaire de Génoprotéomique Appliquée-Cancer (GIGA-Cancer), University of Liege, Belgium; ^2^ Department of Radiotherapy-Oncology, Centre Hospitalier Universitaire (CHU) de Liège, Belgium; ^3^ Laboratory of Myeloid Cell Immunology, VIB, Brussels, Belgium; ^4^ Laboratory of Cellular and Molecular Immunology, Vrije Universiteit Brussel, Brussels, Belgium; ^5^ Systems and Modeling (GIGA-Systems Biology and Chemical Biology), University of Liège, Belgium; ^6^ GIGA Bioinformatics Platform, University of Liège, Belgium

**Keywords:** neoadjuvant radiotherapy, tumor microenvironment, tumor surgery, lung metastases, NK cells

## Abstract

Neoadjuvant radiotherapy (neoRT) used in cancer treatments aims at improving local tumor control and patient overall survival. The neoRT schedule and the timing of the surgical treatment (ST) are empirically based and influenced by the clinician's experience. The current study examines how the sequencing of neoRT and ST affects metastatic dissemination. In a breast carcinoma model, tumors were exposed to different neoRT schedules (2x5Gy or 5x2Gy) followed by surgery at day 4 or 11 post-RT. The impact on the tumor microenvironment and lung metastases was evaluated through immunohistochemical and flow cytometry analyses.

After 2x5Gy, early ST (at day 4 post-RT) led to increased size and number of lung metastases as compared to ST performed at day 11. Inversely, after 5x2Gy neoRT, early ST protected the mice against lung metastases. This intriguing relationship between tumor aggressiveness and ST timing could not be explained by differences in classical parameters studied such as hypoxia, vessel density and matrix remodeling. The study of tumor-related inflammation and immunity reveals an increased circulating NK cell percentage following neoRT as compared to non irradiated mice. Then, radiation treatment and surgery were applied to tumor-bearing NOD/SCID mice. In the absence of NK cells, neoRT appears to increase lung metastatic dissemination as compared to non irradiated tumor-bearing mice.

Altogether our data demonstrate that the neoRT schedule and the ST timing affect metastasis formation in a pre-clinical model and points out the potential role of NK cells. These findings highlight the importance to cautiously tailor the optimal window for ST following RT.

## INTRODUCTION

Radiotherapy (RT) is a standard treatment used for at least 50% of cancer patients. For long, RT was given as daily low doses during multiple weeks (normofractionated RT). More recently, technological advances allowed to more precisely target radiation to the tumor, enabling the delivery of high doses in fewer fractions (hypofractionated RT). RT is used either alone (curative RT) or prior to surgery as neoadjuvant radiotherapy (neoRT). The latter improves local tumor control and patient overall survival compared to surgery alone [1, 2]. In the case of locally advanced rectal cancer (LARC), neoRT decreases the risk of local recurrence by more than 60% compared to surgery alone. However, it has no or little impact on patient overall survival and on the occurrence of distant metastases [3]. Intriguingly, two independent groups showed that the timing of surgery after neoRT affects patient overall survival [4, 5]. One of these trials identified that patients operated within 5 days following RT had a worse overall survival and disease-free survival compared to those patients submitted to curative surgery after a treatment-free window of more than 5 days [5]. However, no difference in local control was observed between the two groups. These alarming observations suggest that the timing of surgery treatment (ST) might influence metastasis occurrence and patient overall survival after neoRT. In clinical practice, the selection of surgery timing is based on the aim to downsize the tumor to avoid positive margins during surgery, as well as on the risk of treatment side effects and of cancer cell repopulation after treatment [6]. Currently, the trend is to lengthen the time between the neoRT and the surgery in order to administer other neoadjuvant treatment. However, none of the main international clinical studies conducted on neoRT addressed the impact of surgery timing on metastatic dissemination [6, 7].

A tumor is composed of cancer cells, non-cancer cells (inflammatory, endothelial and fibroblastic cells) and extracellular matrix [8], which all together elaborate a specific tumor microenvironment that influences the tumor phenotype [9]. Ionizing radiations (IR) target both cancer cells and their microenvironment that may in turn influence the tumor aggressiveness [10]. Some studies have reported the IR influence on tumor aggressiveness. One has to admit that patient cohorts, treatments applied (e.g. dose and fractionation) and animal models used were highly heterogeneous [11, 12]. IR can affect the microenvironment through different ways including a modulation of angiogenesis, hypoxia, inflammation or extracellular matrix remodeling and subsequently the risk of tumor metastases [13-17]. Obviously, these parameters are not static and evolve during and after RT. When clinical observations were published [5], the authors hypothesized that the tumor microenvironment after neoRT evolves in time, providing either a “good” or a “bad window” for surgery that could affect or not tumor dissemination. Moreover, we postulate that the RT schedule (i.e. the dose per fraction and the treatment duration) could also influence the tumor microenvironment. In order to address the impact of neoRT and ST schedules on metastatic occurrence, in a rational way, we developed a pre-clinical model of breast cancer that reproduces neoRT and ST protocols. The modifications occurring in the tumor microenvironment were examined at the time of ST after different RT schedules and at different surgery timing in order to define the tumor microenvironment status during the surgical procedure. This pre-clinical approach provides unprecedented data on the impact of neoRT and ST schedules and draws the attention of clinicians on the existence of an optimal window for ST after neoRT.

## RESULTS

### Delaying the surgery after hypofractionated neoRT decreases lung metastasis formation

**Figure 1:**
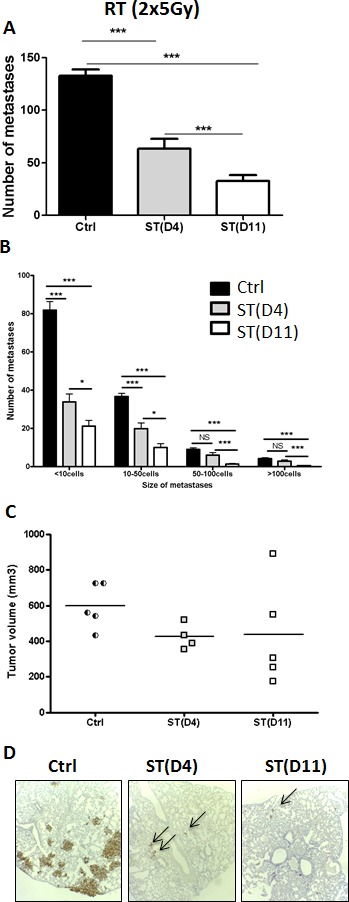
Impact of the timing of surgery after hypofractionated neoRT on lung metastases compared to non irradiated control mice Control SCID mice (ctrl) did not received neoRT prior to surgery. For irradiated SCID mice, tumors were resected (surgery therapy: ST) at day 4 (D4) or 11 (D11) post-RT. **A.** Average number of global lung metastases. **B.** Stratification of lung metastasis number according to the size of metastatic foci (< 10 cells; 10 to 50 cells; 50 to 100 cells and >100 cells). **C.** Tumor volume (mm³) at the time of surgery. **D.** Representative sections of lungs collected at the end of the experience. Metastatic cells were labeled with an anti-human Ki67 antibody (4x Magnification). The arrows delineate representative metastatic foci. Results are expressed as mean + SEM. *p < 0.05. **p < 0.01 ***p < 0,001; ns = non statistically significant.

We first studied the impact of the timing of surgery after hypofractionated neoRT (2x5Gy) on lung metastases. Surgical tumor resection was performed at two time points (early ST at D4 and late ST at D11) chosen according to clinical observations, in which the ST timing has been demonstrated to have a pivotal role for patient overall survival [4, 5]. Mice were sacrificed 45 days after the beginning of the RT, so that micro-metastases had time to develop as previously described [18]. The global number of metastases was determined through IHC analyses performed on lung sections (Figure 1A). Lung metastases were also stratified according to the number of cancer cells by metastatic foci: <10 cells, 10 to 50 cells, 50 to 100 cells and >100 cells (Figure 1B) because in clinic, the size of metastases has a direct biological impact.

Hypofractionated (2x5Gy) RT drastically reduced the global number of lung metastases (Figure 1A), as well as their size (Figure 1B). Notably, the number of metastases was higher when ST was performed 4 days after hypofractionated RT, as compared to that performed at 11 days. This observation was confirmed by the stratification of metastatic foci according to their size (Figure 1B). It is worth noting that the tumor volumes at the time of surgery were similar in all experimental groups (Figure 1C). Furthermore, no correlation was established between the tumor volume reached at surgery and the number of metastases (the linear regression coefficient (r²) was 0.18 (*p* = 0.58) in control group, and 0.003 (*p* = 0.93) and 0.67 (*p* = 0.08) in mice subjected to early and late ST, respectively). No excess of mortality was observed between groups.

To determine how the status of the tumor microenvironment at the time of surgery could influence the metastatic dissemination, we next evaluated different parameters that could affect the tumor phenotype. Immunohistochemical stainings (IHC) were performed to determine cell proliferation rate (Ki67), blood vessel density and size (CD31) and hypoxia (pimonidazole). As expected, computerized quantifications revealed higher necrotic and hypoxic areas following hypofractionated neoRT as compared to non-irradiated control tumors (Supplemental Figure 1A-C). The density of blood vessels assessed by CD31 staining was similar in all experimental groups, together with the density of proliferating cells (Ki67^+^ cells) (Supplemental Figure 1D-H). An extensive extracellular matrix remodeling associated with cancer progression relies on the activity of several proteases including serine and metalloproteases (MMP). The expression of several proteases (MT1-MMP) or inhibitors (TIMP-1, TIMP-2 and PAI-1) determined by RT-PCR was not modulated by the experimental conditions (Supplemental Figure 1I-L).

**Figure 2:**
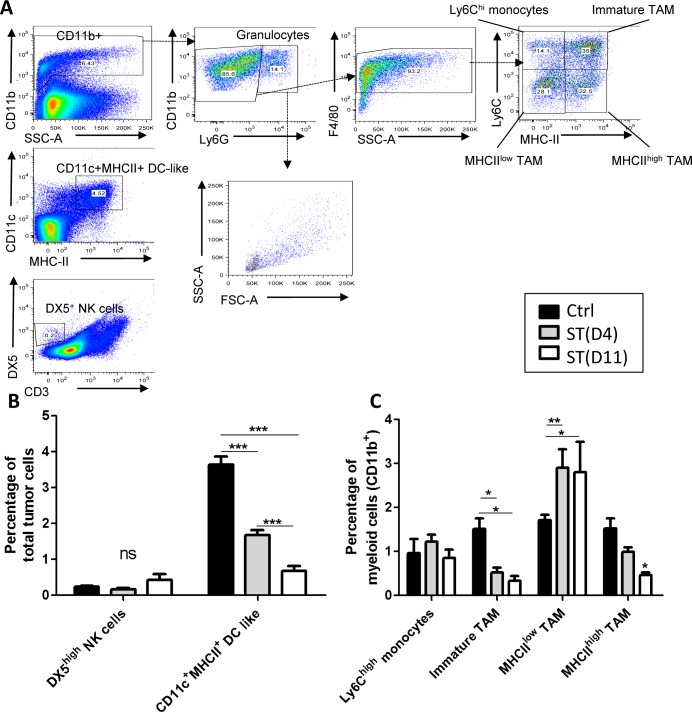
FACS analysis of cells isolated from primary tumors subjected to hypofractionnated RT Control SCID mice received only ST. Irradiated SCID mice received 2x5Gy neoRT and tumors were collected 4 (D4) or 11 (D11) days after the end of RT. Single-cell suspension was prepared from primary tumors at the time of surgery and stained for the indicated markers. **A.** Gating strategy for FACS data analyses. **B.** Percentage of NK and dendritic Cells of total number of tumor cells. And **C**. Percentage of Ly6C^high^ monocytes, immature TAMs, MHCII^high^ and MHCII^low^ TAM of myeloid cells. (*n* = 5-6) *p < 0.05 ; **p < 0.01; ***p < 0.001.

We next performed FACS analysis to study the different subtypes of innate immune cells infiltrating the tumor or circulating in the blood, at the time of surgery. Inside the tumor, myeloid cells represent about 7.5% of the total cells composing the tumor. The proportion of F4/80^+^ TAM represents around 70% of the total number of CD11b^+^ cells in all groups. A significant decrease of immature TAM (represented in percentage of CD11b^+^ cells in the tumor) was observed following hypofractionated neoRT as compared to non-irradiated control tumors, with no impact of ST timing (Figure 2). Interestingly, we observed a significantly higher proportion of MHCII^low^ proangiogenic TAM and a significant decrease of MHCII^high^ prometastatic TAM following hypofractionated neoRT as compared to control mice. These data suggest a switch from MHCII^high^ to MHCII^low^ TAM following ionizing radiation. However, ST timing did not affect this shift. The percentage of neutrophils was not significantly different between experimental groups (data not shown). In sharp contrast, the percentage of CD11c^+^ MHC-II^+^ dendritic cells (DC like) was smaller after neoRT compared to non-irradiated mice. Interestingly, late surgery after neoRT (at D11) led to a two-fold reduction of DC-like cell percentage and this was associated with decreased lung metastases (0.67% ± 0.25 at D11 *versus* 1.67% ± 0.37 at D4) (Figure 2C). There was no significant difference in DX5^high^ NK cells (0.25% ± 0.17) (Figure 2C).

**Figure 3:**
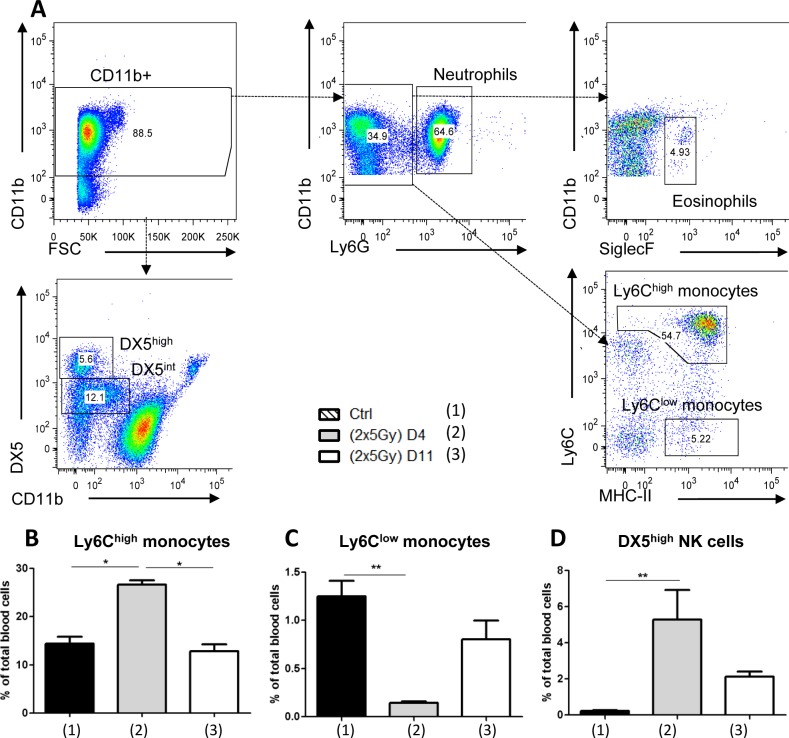
FACS analysis of total blood cells in SCID mice subjected to hypofractionated RT Mice (*n* = 5-6) were irradiated or not (ctrl mice) with 2x5Gy neoRT. Blood was collected 4 (D4) or 11 (D11) days after the end of RT. Blood cells were isolated and stained for the indicated markers. **A.** Gating strategy for FACS data analyses according to several markers used and FSC (Forward Scatter). (B-D) Percentages of Ly6C^high^ **B.** and Ly6C^low^ monocytes **C.**, and NK cells **D.** of total blood cells *p < 0,05; **p < 0,01; ***p < 0.001.

Regarding circulating innate immune cells (Figure 3), eosinophils represent a small cell population (< 1.68%), while neutrophils cover about 50% of total blood cells. Such a cell distribution was not affected by treatment. We also analyzed circulating Ly6C^low^ patrolling monocytes and Ly6C^high^ inflammatory monocytes, the latter being known to be rapidly and massively recruited during inflammation [19]. A similar proportion of these two monocyte subtypes was detected in non irradiated mice and at day 11 post-hypofractionated neoRT. Higher Ly6C^high^ and lower Ly6C^low^ monocyte proportions were seen 4 days after hypofractionated neoRT. Nevertheless, these differences in monocyte distribution were not related to the metastatic status. Intriguingly, although very few DX5^high^ NK cells were detected in the blood of control mice (0.22% ± 0.12 of total blood cells), neoRT induced a drastic increase of circulating DX5^high^ NK cells in mice subjected to tumor resection at day 4 (5.27% ± 4) or at day 11 (2.12% ± 0.58). The extent of this enhancement in circulating NK cell percentage was associated with the increased metastatic phenotype observed after early ST.

### The timing of surgery slightly modulates lung metastases after normofractionated neoRT

**Figure 4:**
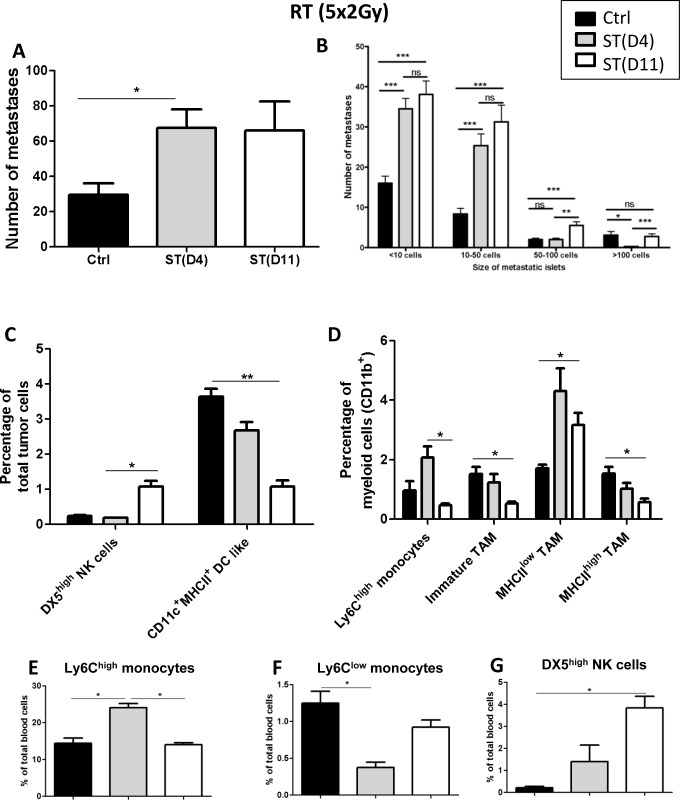
Impact of the timing of surgery after normofractionated (5x2Gy) neoRT on lung metastases Control SCID mice (ctrl) did not received neoadjuvant RT prior to surgery. For irradiated SCID mice, tumors were resected (surgery therapy: ST) at day 4 (D4) or 11 (D11) post-RT. **A.** Average number of global lung metastases. **B.** Stratification of lung metastasis number according to the size of metastatic foci (< 10 cells; 10 to 50 cells; 50 to 100 cells and >100 cells). Results are expressed as mean + SEM. *p < 0.05. **p < 0.01 ***p < 0,001; ns = non statistically significant. (C-G) FACS analyses of tumor and blood samples (*n* = 5-6). The gating strategies are described in figures 2 and 3. **C.** Percentage of NK and dendritic cells. **D**. Percentage of Ly6C^high^ monocytes, immature TAMs, MHCII^high^ and MHCII^low^ TAM. (E-G) Percentages of Ly6C^high^ **E.** and Ly6C^low^ monocytes **F.**, and NK cells **G.**. *p < 0,05; **p < 0,01; ***p < 0.001.

Surprisingly, mice subjected to normofractionated (5x2Gy) neoRT displayed an increased number and size of metastases as compared to non irradiated control mice (Figure 4A, 4B). In these conditions, the ST timing did not affect the global number of lung metastases. This pro-metastatic effect of RT could not be ascribed to a failure to reduce tumor growth. Indeed, the potent anti-tumor effect of 5x2Gy RT was demonstrated by the drastic inhibition of tumor growth seen in a kinetic study (Supplemental Figure 2). The tumor volume remained stable 3 weeks after RT, while it increased 3-fold in control non irradiated mice. As expected, neoRT induced tumor necrosis (Supplemental Figure 2).

Although ST timing did not affect the global number of metastases, a stratification of metastases showed that delaying the surgery slightly increased the number of large metastatic foci (> 50 cells) (Figure 4B). The tumor volumes at the time of surgery were similar in all experimental groups (Supplemental Figure 3).

In this experimental setting, we confirmed the impact of neoRT on the innate inflammatory cell profile, both in tumor (Figure 4C, 4D) and blood (Figure 4E-4G) samples. Importantly, DX5^high^ NK cell percentage was again drastically increased by neoRT in blood samples. The proportion of NK cells was 2-fold higher in mice subjected to late ST (day 11), in which larger metastatic foci were observed. (Figure 4G).

### Impact of NK cells on lung metastases formation after neoRT

**Figure 5:**
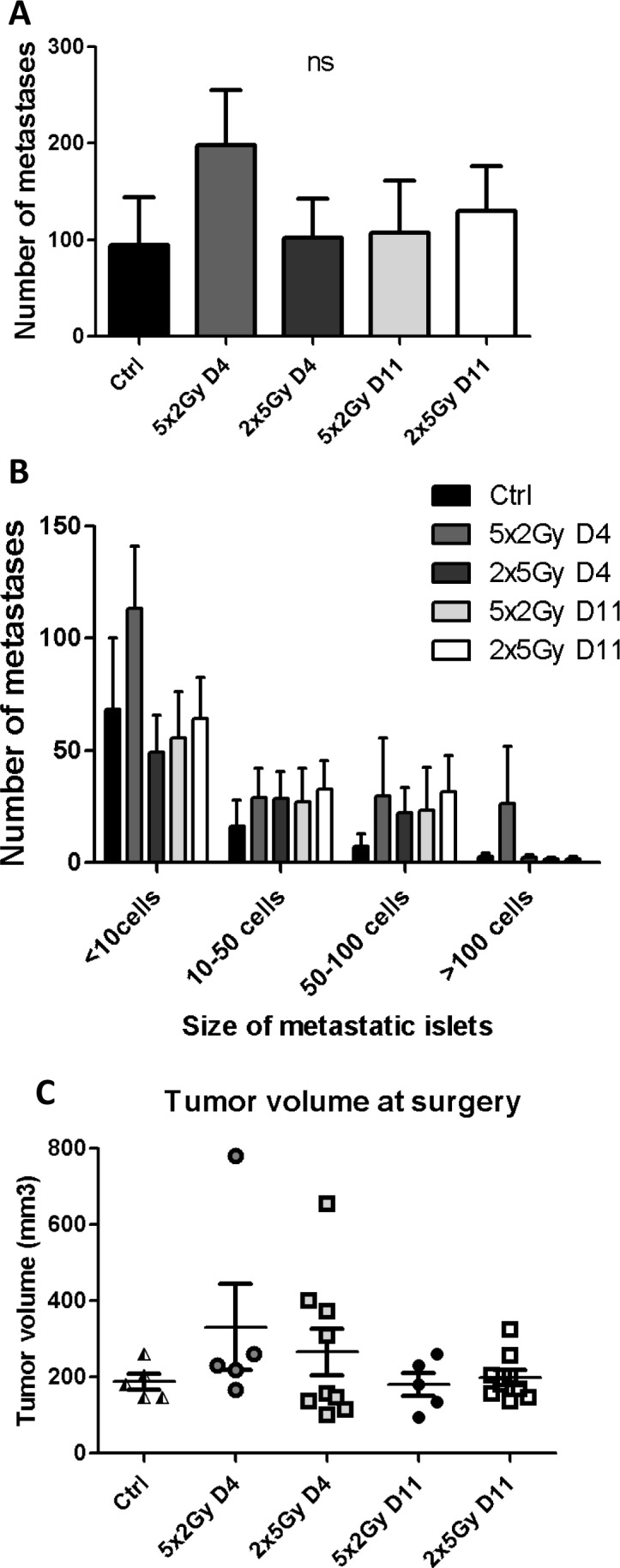
Impact of neoRT and ST timing on lung metastases in NOD/SCID mice Tumors implanted into NOD/SCID mice were resected at day 4 (D4) or at day11 (D11) after normofractionated RT (5x2Gy) or hypofractionated RT (2X5Gy). Control NOD/SCID mice (ctrl) did not received neoRT prior to surgery (n = 4-9). **A.** Average number of global lung metastases. **B.** Stratification of lung metastasis number according to the size of metastatic foci (< 10 cells; 10 to 50 cells; 50 to 100 cells and >100 cells). **C.** Tumor volume at the time of tumor resection. Results are expressed as mean + SEM. *p < 0.05; **p < 0.01; ***p < 0.001; ns = non statistically significant.

We next postulated that the kinetics of NK cell recruitment following neoRT could influence the metastatic occurrence. To address this crucial issue, we used mature NK cell-deficient NOD/SCID mice and compared the effect of RT treatments (i.e. normo- *vs* hypo-fractionated RT) at different ST timings (Figure 5). In these NOD/SCID mice, the number of metastases (both global and large metastatic foci) was similar in control non-irradiated mice and in neoRT-treated mice, independent of ST timing (Figure 5). The reduction of metastatic occurrence in mice subjected to hypofractionated neo-RT and late ST observed in SCID mice (Figure 1) was not seen anymore in NOD/SCID mice (Figure 5). These data underline the importance of NK cells in the regulation of the metastatic phenotype following neo-RT.

## DISCUSSION

In this work, we hypothesized that both the surgery timing (ST) and the neoRT schedule, could affect the metastatic dissemination of cancer cells. We think that the impact of the surgery on metastatic spreading could be modulated by the temporal evolution of tissue remodeling induced by RT. We demonstrate that neoRT influences the recruitment of innate inflammatory cells in blood and in tumors, influencing the optimal window for tumor resection. Mechanistically, the pivotal role of NK cells is supported by the failure of neoRT and ST schedules to impact metastases formation in NK-deficient mice (NOD-SCID).

Herein, we are providing an unprecedented *in vivo* experimental tool for studying the impact of neoRT and ST on the primary tumor microenvironment and metastases. The strength of our model relies on the capacity (i) to locally irradiate several times the tumors as performed in clinical practice and (ii) to perform surgery at different time points after the end of RT without a rapid regrowth of the tumor. It is worth noting that neoRT aims at controlling microscopic disease and therefore it requires lower doses than RT used in a curative purpose. Doses and RT schedules have been adapted to mouse constraints with the objective to be as close as possible to RT schedules used in clinic. Importantly, in this model, mice spontaneously developed moderated lung metastases few weeks after the surgery, reproducing thereby a natural metastatic process. In addition, our model overcomes the limitations of most syngeneic models, which are fast growing tumors that are not compatible with neoRT and surgery before metastatic spreading. Although, a direct impact of RT on primary tumor growth has not been observed at the time of surgery, a blockade of tumor progression was detected at later time points (three weeks post-RT). Furthermore, increased tumor necrotic areas were detected in RT-treated mice.

The most intriguing finding of our work is the demonstration that the ST timing as well as the RT schedule influence the formation of metastases. The impact of ST timing after normofractionated RT (5X2Gy) is quite limited, but larger metastatic islets were observed when surgery was performed at day 11 compared to day 4. In sharp contrast, with hypofractionated RT (2X5Gy), the tumor dissemination was reduced when ST was performed later. Our results are in accordance with clinical observations suggesting a link between the timing of ST and patient overall survival [4, 5].

Several mechanisms could be involved in the metastatic spreading after neoRT. Importantly, there was no difference in tumor cell proliferation, tumor size and local recurrence according to the RT schedule in our experiments. Differences in treatment schedules (2x5Gy within 2 days and 5x2Gy within 5 days) might influence the release of viable cells into the blood flow and subsequently the colonization of lungs. This may partially explain the increase of larger metastases observed when ST was performed at day 11 compared to day 4 after 5x2Gy RT. However, this hypothesis of more viable cell seeding does not fit with the reduced metastasis formation observed after hypofractionated RT and ST performed at day 11. Clinical trials comparing short and long course RT in LARC are facing the same problem of treatment duration [20, 21], but none of these trials achieved to demonstrate the superiority of one treatment compared to the others. Altogether, these observations underline that tumor dissemination after neoRT is complex and depend on multiple interactions between cancer cells and their microenvironment.

The reductionist view of a tumor composed only of cancer cells has remarkably evolved and the implication of the tumor microenvironment during cancer progression is now well established [8]. The various molecular and cellular components of the host compartment (extracellular matrix, fibroblasts, endothelial, inflammatory and immune cells), the metabolic state of the tumor (hypoxia, reoxygenation, Warburg effect) as well as the systemic crosstalk between primary tumor and secondary organs could affect the recruitment of bone marrow-derived cells, the selection of aggressive tumor cells and the migration to secondary organs. The differences in the metastatic spreading observed in our experiments could not be explained by a difference in terms of protease activity, hypoxia or vessel density examined at the time of surgery. These results prompted us to focus on cancer related-inflammation and on the immune system, which are known to play a pivotal role in tumor dissemination [22]. In tumors, both RT schedules were associated with an increase in the MHCII^low^ (M2-like) TAM proportion and a decrease in the MHCII^high^ (M1-like) TAM proportion, suggesting a shift from M1 to M2-like TAM induced by RT. This observation fits with increased RT-induced hypoxia seen in our tumor models, and with the previously reported higher infiltration of hypoxic tumor areas by M2-like TAM [23, 24]. However, no correlation could be established between macrophage polarization at the time of surgery, ST timing, RT fractionation and the propensity to metastasize. The levels of dendritic cells were decreased following RT, independently of the fractionation and without a direct link with the metastatic profile. RT has also been reported to induce the release of tumor-associated antigens and to upregulate immunomodulatory cell surface molecules leading to anti-tumor immunity [25]. Although cytotoxic T cells contribute to this process, tumor cells often downregulate their MHCI expression and escape from T cell-mediated killing. T lymphocytes are unlikely to contribute in our model based on the use of immuno-deficient mice. In this study, flow cytometry analyses pointed out a regulation of NK cell recruitment in the primary tumor and in the blood stream, which was affected by RT schedule. An earlier (at day 4) and higher (3-fold increase) recruitment of NK cells was observed in hypofractionated RT as compared to normofractionated RT. In the latter protocol, similar percentage of NK cells (around 5%) was only reached at day 11. Our study highlights for the first time an impact of RT fractionation on the kinetics of NK cell mobilization.

The functional relevance of this intriguing finding is demonstrated by the use of NOD/SCID mice instead of SCID mice. Notably, neoRT schedules and ST timing failed to influence the metastatic profile in NOD/SCID mice. Therefore, NK cell recruitment/mobilization induced by RT contributes to the modulation of metastases following neoRT and surgery. NK cells have been shown to play a crucial role in mediating tumor clearance following surgery and their anti-tumoral activity is impaired upon surgical stress [26]. Moreover, primary tumor hypoxia compromised NK cell cytotoxicity in the premetastatic niche leading to higher metastatic burden [27]. Altogether these data suggest that RT and ST protocols could affect both the mobilization and the cytotoxic activity of NK cells, influencing thereby the metastatic profile of a tumor. Further studies are required to decipher the exact mechanisms of NK cell regulation by RT and ST. A recent report demonstrates the interest to combine RT with an immunotherapy approach that triggers NK cell immune response [28]. In this study, the sequence of treatments appears crucial, RT being efficient only when applied before immunotherapy [28]. This recent finding combined to our data reflect the complex cascade of events occurring after RT, which has to be further explored to optimize current treatments used in clinical practice [29].

In conclusion, our study provides the first experimental demonstration of the importance of optimizing the time interval between neoRT and surgery. The lowering of the metastatic burden when surgery was performed later after hypofractionated (2x5Gy) neoRT is consistent with the current trend to lengthen the time between neoRT and surgery in clinic. This lengthening of the interval between RT and ST will allow the administration of others adjuvant treatment modalities (i.e. immunotherapy, chemotherapy…) and is under study, for example, in the case of rectal cancer [30]. The mechanisms underlying the metastatic dissemination following treatments are not fully understood and probably rely on a complex and dynamic mosaic of cellular and molecular interactions between cancer cells and their tumor microenvironment, in which NK cells appear as key actors.

## MATERIALS AND METHODS

### Cell culture

Human breast cancer MDA-MB-231 cells (clone C14) were used as previously described [18]. Cells were grown in Dulbecco's Modified Eagle's Medium (DMEM) supplemented with 10% Fetal Bovin Serum, L-glutamine (2mM) and penicillin (100U/ml)-streptomycine (100μg/ml). All culture reagents were purchased from Gibco-Life Technologies (Invitrogen Corporation, Paisley, Scotland).

### Mice

Female 6-8 weeks old SCID mice (Charles River, France) or NOD/SCID (Animalerie centrale of University of Liège) were maintained at the “Animalerie Centrale” of the University of Liège in a confined area. All the experiments were performed in accordance with the ethical committee of the University of Liège.

### Tumor xenograft model

Cancer cells were trypsinized and resuspended in serum-free DMEM (1x10^6^ cells/200μl). A mixture (400μl) of Matrigel and cell suspension (1:1) was injected subcutaneously in the flank of SCID mice as established in our laboratory and previously described [18, 31]. When the tumor volume reached 400mm³, mice were randomly assigned in the different treatment groups.

### NeoRT treatment

Tumor bearing mice were locally irradiated with either 10 Gy in 5 fractions (normofractionated RT: 5x2Gy) or 10 Gy in 2 fractions (hypofractionated RT: 2x5Gy) administered daily. The RT was performed with an orthovoltage x-ray (Stabilipan, Siemmens) using a filter Cu 0,5 mm, dose rate 0,265 Gy/min at DSP 40 cm, 150 Kv and the dose was prescribed at 5mm deepness. Mice were immobilized in a 2 mm thick plexiglass tube and sedated with 50μg/Kg of medetomidin hydrochlorid (Domitor, Orion Pharma). The effect was reversed with 1mg/Kg of atipamezol hydrochlorid (Antisedan, Orion Pharma) directly after irradiation. Cerrobend shielding block was used for protecting mice and organs at risk against irradiation. A 18 mm hole in the block allowed to specifically irradiate the tumor and the possible surrounding microscopic invasion of tumor cells.

### Surgical tumor resection and metastases quantification

Tumor bearing mice were operated the 4th (D4) or 11th day (D11) after the end of the RT treatment or when tumor volume reaches 400mm³ under anesthesia with xylazine (75mg/kg, VMD, Anedonk, Belgium) and ketamin (10mg/kg, CEVA, Brussels, Belgium). Tumors were carefully removed and surgical resection included a margin of 3 mm of healthy tissue. Skin was suturated with 5-0 silk (Perma-hand, Ethicon). Tumor fragments were formol-fixed and paraffin-embedded or frozen in air phase of liquid nitrogen for protein and RNA extractions. For hypoxic area detection, mice were injected intraperitoneally with 100 mg/Kg of Pimonidazole (Hydroxyprobe-1, Chemicon) one hour before tumor resection. After surgery, mice were kept alive until D45 [18]. At sacrifice, lungs were formol-fixed and paraffin-embedded. Six lung sections of 5 μm, spaced by 10 sections of 5 μm, were immunostained with an antibody against human Ki67 as previously described [18]. Metastases were manually counted and classified according to their size (<10 cells, 10 to 50 cells, 50 to 100 cells, >100 cells).

### Immunohistochemistry (IHC), image processing and computerized quantifications

Slides (5 μm thick) were autoclaved in Target Retrieval Solution (Dako, S1699, Glostrup, Denmark), incubated in Proteinase K (S3004, Dako) or with EDTA-buffer (Prosan) according to the immunolabelling for Ki67, CD31, and Pimonidazole, respectively. Endogenous peroxidases were blocked by 3% H_2_O_2_/H_2_O (Merck) for 20 minutes, and nonspecific binding was prevented by incubation in PBS/Bovine Serum Albumin 10% (Fraction V, Acros Organics, NJ). Tumor sections were incubated with a mouse monoclonal anti-human Ki-67 antibody (1/100) (clone MIB-1, M7240; DAKO), a rat anti-CD31 antibody (1/100) (Ab56299, Abcam), or a mouse monoclonal anti-pimonidazole antibody (1/50) (Hydroprobe-1 MAb-1 clone 4.3.11.3). After 3 washes in PBS or Tris-HCl for CD-31 staining, slides were incubated with a HRP-conjugated secondary antibody, after post antibody blocking (DPVB Blocking, Immunologic NL) for pimonidazole staining, and revealed with Vector DAB (SK-4100, Vector Laboratories, Burlingame, CA, USA). Slides were counterstained with haematoxylin.

### Image processing and computerized quantification

Immunostained sections were scanned using the digital slide scanner NanoZoomer 2.0-HT system at 0.46 μm/pixel (20X) scanning resolution (Hamamatsu, Mont-Saint-Guibert, Belgium) and images were registered in the RGB (red, green, bleu) color space. Necrotic and stromal areas were eliminated by using the Cytomine web software [32] using the hybrid human-computer approach described previously [33]. For blood vessel and macrophage quantification within tumor regions of interest, image processing and measurements were performed using the toolbox of image analysis of the MATLAB (R2013a) software (Mathworks, Inc.) according to the algorithms described previously [34]. Importantly, binary images resulting from the image processing were systematically compared visually with the corresponding original images and when very occasionally automatic feature detection was not accurate, the threshold was adapted manually. The results are expressed as density defined as the measured area occupied by vessels or positive cells divided by the total area of the corresponding tumor regions of interest.

### Blood lysis and tumor dissociation for FACS analysis

At mice sacrifice, blood was collected through cardiac puncture with heparinized 1ml syringe and 27G needles. Lysis buffer was added on ice, incubated for 6 minutes and neutralized with RPMI medium (Gibco). After 10 minutes centrifugation at 2000 rpm, supernatant was discarded and lysis step was repeated until red blood cells were removed. Cells were suspended in HBSS (Gibco) supplemented with 0.5% heat-inactivated fetal calf serum and 2 mM EDTA. Tumors were treated with 10 U/mL collagenase I, 400 U/mL collagenase IV, and 30 U/mL DNase I (Worthington). Density gradients (Axis-Shield) were used to remove debris and dead cells as described in [23]. Single cell suspensions were incubated with an antibody cocktail and analyzed with FACS Canto II and FACS Diva Software as described in Laoui et al [24].

### Statistical analysis

Statistical analysis for computerized quantification was performed with the statistic toolbox of the Matlab (R2013a) software (Mathworks, Inc.) and Mann–Whitney test was used. For the other experiments (metastasis quantification, PCR, FACS), statistical analyses were carried out using the Prism 5.0 software (GraphPad, San Diego, CA) and we performed unpaired t-test or Mann-Withney test and ANOVA followed by Bonferonni post-test when requested or Kruskal-Wallis test followed by Dunns test when requested. Results were considered significant for *p* < 0.05 and expressed as means ± standard error of the mean (SEM). * : *p* < 0.05; ** : *p* < 0.01; *** : *p* < 0.001.

## SUPPLEMENTARY MATERIAL FIGURES AND TABLE


