# Supplementary material for: The timing of surgery after neoadjuvant radiotherapy influences tumor dissemination in a preclinical model
Source: Oncotarget. 2015 Sep 30;6(34):36825–37. doi: 10.18632/oncotarget.5931 (PMC4742213; doi:10.18632/oncotarget.5931)
Supplement: Supplementary file 1 [file oncotarget-06-36825-s001.pdf]

The timing of surgery after neoadjuvant radiotherapy influences tumor dissemination in a preclinical model

Supplementary Material

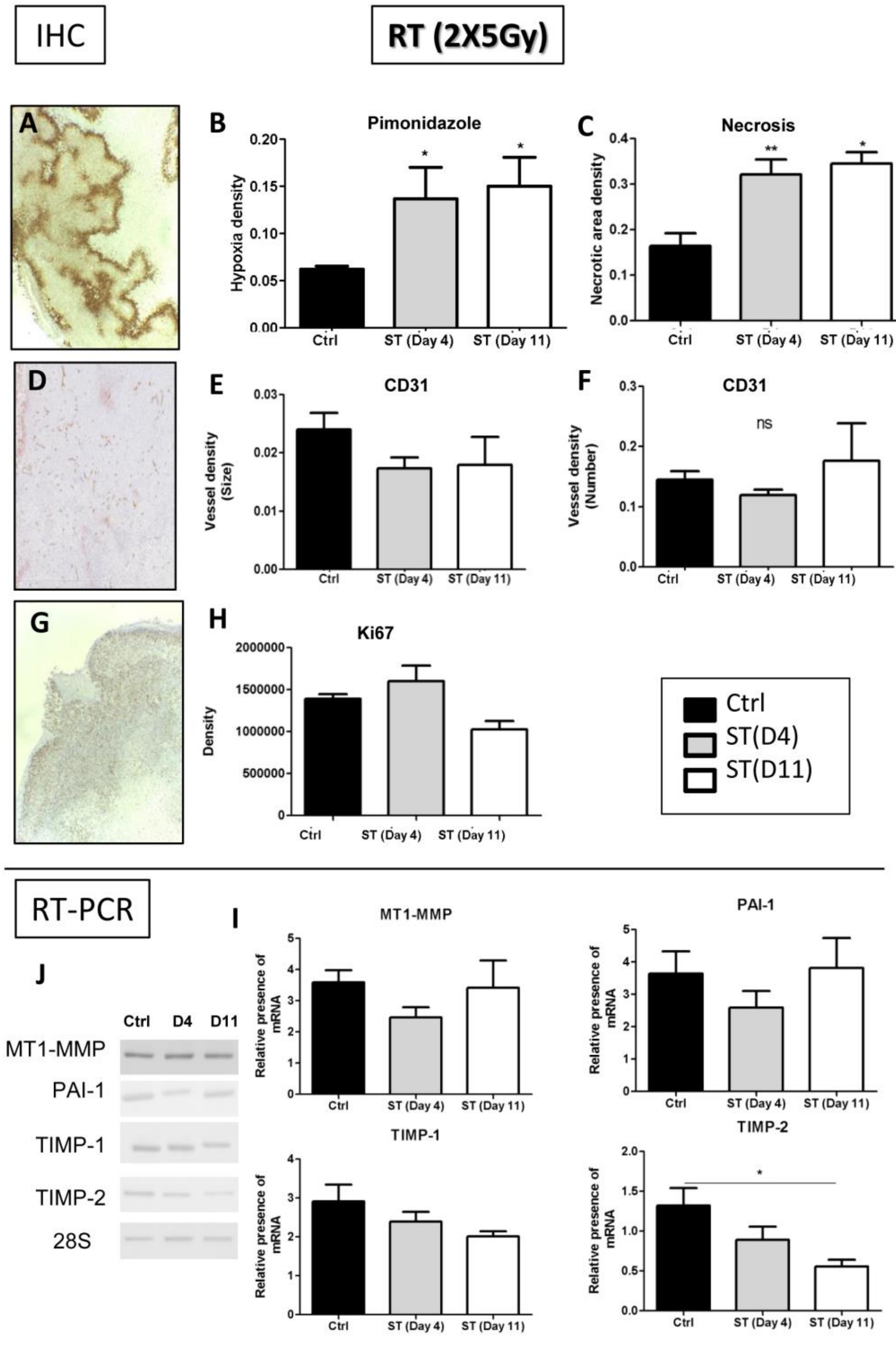

Supplemental figure 1: Immunohistochemical and RT-PCR analyses performed on primary tumors. Tumors implanted into SCID mice were resected at day 4 (D4) or at day11 (D11) hypofractionated RT (2X5Gy). Control SCID mice (ctrl) did not received neoRT prior to surgery (n=5). (A) Immunohistochemical staining of pimonidazole. (B-C) computer assisted quantification of hypoxic area density (B) and necrotic area (C). (D-F) Immunohistochemical staining of CD31 (D) to visualize blood vessels and the corresponding quantification of blood vessel density (E) and number (F). (G, H) Immunohistochemical staining of Ki67 (G) and its quantification (H). (I-J) mRNA samples collected from tumor extract were subjected to RT-PCR. Amplification products were separated by electrophoresis, representative bands are shown in (I) and quantified (J) for MT1-MMP, PAI-1, TIMP-1 and TIMP-2 relatively to 28S. 28S was used as a loading control.

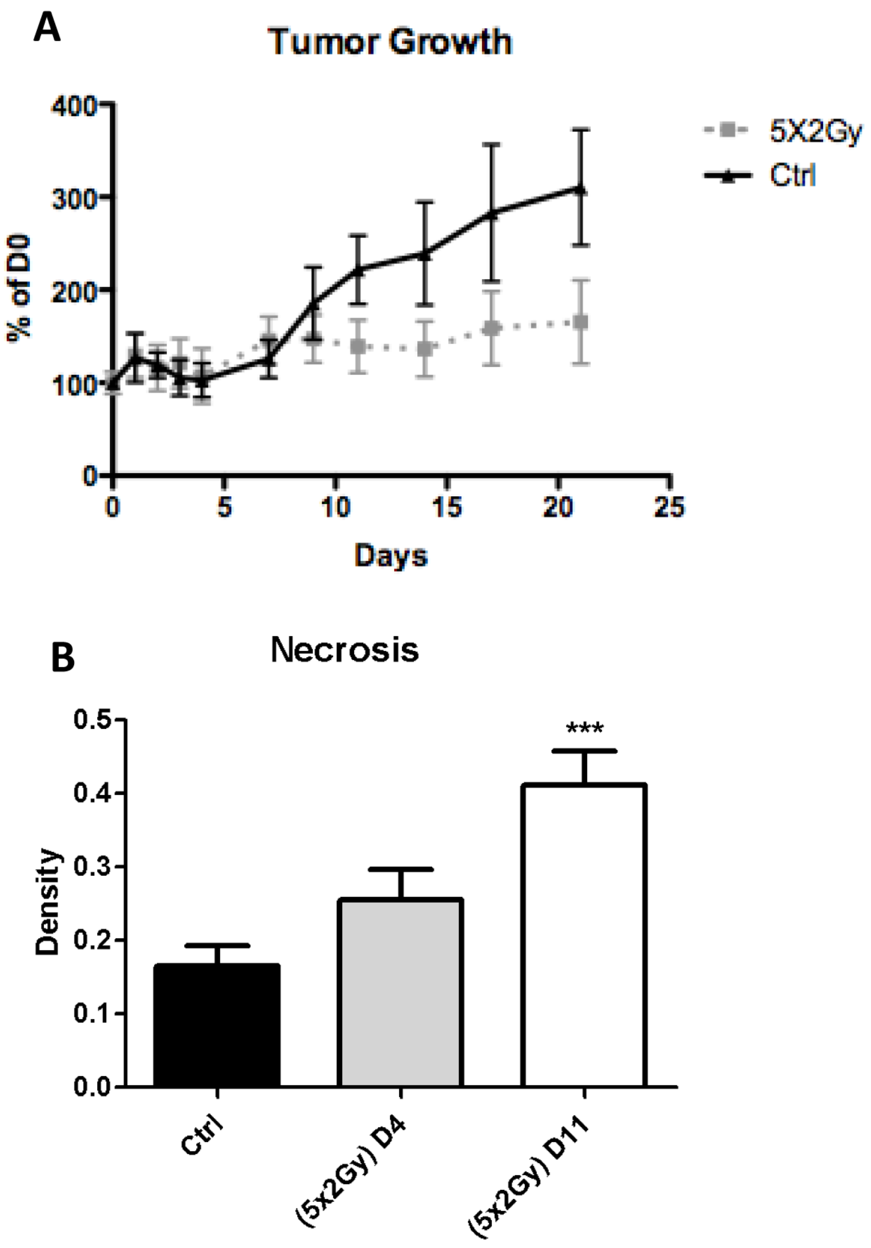

Supplemental Figure 2: Impact of normofractionated (5x2Gy) neoRT on tumor growth. (A) Tumor growth curves of non-irradiated control SCID mice (Ctrl) or SCID mice subjected to normofractionated (5x2Gy). Results are expressed as percentage of tumor volume at the first day (D0) of RT. (B) Computer assisted quantification of necrotic areas detected on histological slides.

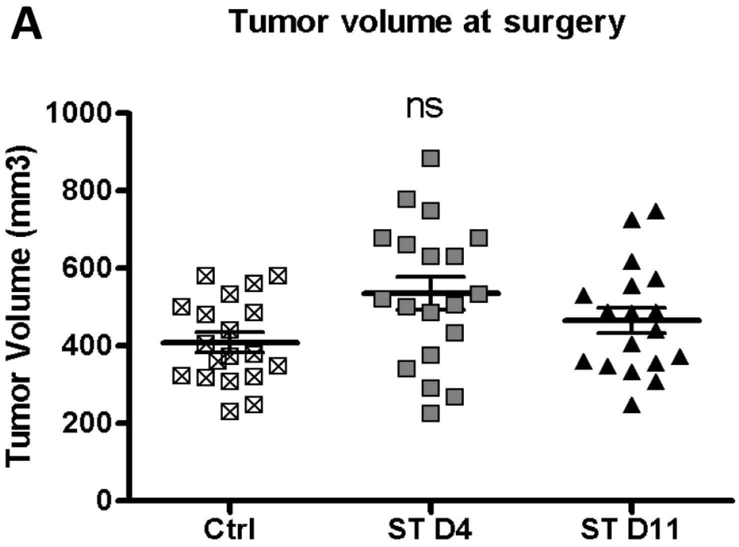

Supplemental Figure 3: Tumor volume reached at the time of surgery after normofractionated RT. SCID mice were either irradiated (5X2Gy) or not (Ctrl). Surgery (ST) was performed 4 or 11 days after RT. The tumor volumes presented correspond to the experiment described in figure 4.

Supplementary table 1: Primers used for RT-PCR (Supplemental Figure 1) with GeneAmp Thermostable rTth enzyme (Applied Biosystems) after RNA extraction using the High Pure RNA Isolation Kit (Roche Diagnostics).

**Gene of interest**	**Forward primer**	**Reverse primer**
28S	GATTCTGACTTAGAGGCGTTCAGT	GTTCACCCACTAATAGGGAACGTGA
MT1-MMP	GGATACCCAATGCCCATTGGCCA	CCATTGGGCATCCAGAAGAGAGC
TIMP-1	CATCCTGTTGTTGCTGTGGCTGAT	GTCATCTTGATCTCATAACGCTGG
TIMP-2	CTCGCTGGACGTTGGAGGAAAGAA	AGCCCATCTGGTACCTGTGGTTCA
PAI-1	AGGGCTTCATGCCCCACTTCTTCA	AGTAGAGGGCATTCACCAGCACCA
